# Questionable research practices of medical and dental faculty in Pakistan – a confession

**DOI:** 10.1186/s12910-024-01004-4

**Published:** 2024-01-31

**Authors:** Ayesha Fahim, Aysha Sadaf, Fahim Haider Jafari, Kashif Siddique, Ahsan Sethi

**Affiliations:** 1 University College of Dentistry, The University of Lahore, Lahore, Pakistan; 2 Shifa College of Dentistry, Shifa Tameer-e-Millat University, Islamabad, Pakistan; 3 Azra Naheed Dental College, Superior University, Lahore, Pakistan; 4 QU Health, Qatar University, Doha, Qatar

## Abstract

**Purpose:**

Intellectual honesty and integrity are the cornerstones of conducting any form of research. Over the last few years, scholars have shown great concerns over questionable research practices (QRPs) in academia. This study aims to investigate the questionable research practices amongst faculty members of medical and dental colleges in Pakistan.

**Method:**

A descriptive multi-institutional online survey was conducted from June-August 2022. Based on previous studies assessing research misconduct, 43 questionable research practices in four domains: Data collection & storage, Data analysis, Study reporting and Collaboration & authorship were identified and investigated. Descriptive (Frequencies, Percentages, Mean, SD) and Inferential (chi square) statistics were calculated.

**Results:**

A total of 654 faculty members responded. Every respondent reported committing at least one QRP in their career. The most common QRPs included deliberately failing to mention funding, publishing program evaluation data not meant for research purposes or approved by an ethical body, inappropriately storing identifiable information and non-disclosure of any conflicts. There was significant association of age, gender and academic rank with QRPs in ‘Data collection and storage’ and ‘Data Analysis’ domains.

**Conclusion:**

Medical and dental faculty members participating in this study are involved in a range of questionable research practices (QRPs) in Pakistan. Their confession might have contributed to the faculty developing self-awareness and reinforcing academic integrity. There is a need for reviewing policies and practices to improve research culture. Future research should explore the factors resulting in such practices.

## Introduction

Research in healthcare has gained increasingly high value in the society over the last few decades [1]. Good quality research by health professionals not only leads to significant discoveries in disease management and patient care [2], it contributes substantially to the improvement of health professional education system [3]. Intellectual honesty and integrity are the cornerstones of conducting any form of research. The foundation of sound scientific practice dictates that research must be conducted in a responsible manner [4]. The origin of ‘Ethics in Research’ stems back to 4th century BC with the Hippocratic oath followed by Adab-al-Tabib in the 9th century [5]. In 1752, Royal Society of London was the first to initiate peer review process for ethical research practice [6]. In the 19th century, ethics and good practice in research evolved exponentially with the introduction of Declaration of Helsinki in 1964 [7], the National Research Act of 1974 [8], the FDA guidelines [9], ICMJE guidelines and many more. The ethical guidelines are still being updated regularly by many governing bodies like World Health Organization (WHO), National Institute of Health (NIH), General Medical Council (GMC) [10] etc. Despite the available recommendations, many a times we witness irresponsible literary work.

Scholars have shown major concern over questionable research practices. Journal editors have emphasized how research misbehaviours pose a threat to academic integrity and validity of research [11]. Editors have noticed several incidences of ‘self-citation and self-plagiarism’ [12], ‘salami-slicing’ of one research [13] and shady authorship practices [14]. In 2014, Journal editorial reported that a lot of papers submitted to the Journal of Orthodontics do not have ethical approval letters [15]. A study reported that 13% of the papers published in 2013 lacked ethical approval letters [16]. There is fair amount of work published on research misconduct in social and natural sciences [17], applied linguistics [18], business research [19], management sciences [20], psychology [21], communication research [22]. Recently, QRP in health professionals in USA, Canada and Europe were recorded in a self-perceived questionnaire [23]. However, such reports are not available for low-economic countries like Pakistan.

Irresponsible research practices not only include deliberate misconduct e.g., falsification, fabrication and plagiarism, but questionable research practices (QRPs) as well [24]. In health professional education (HPE), QRPs have been described as actions involving inappropriate research design, poor data management, inadequate respect for study participants, unjustifiable authorship or publishing practices, negligence in data observation and analysis and carelessness in reviewing and editing [25]. Whereas practices such as plagiarism and fabrication of data are clearly deliberated as “fraud” according to WHO and NIH guidelines, QRPs exploit the gray area of ethics and are free of socially stigmatized behaviour [26]. As a consequence, QRPs have become more prevalent and cause damage to the academic enterprise in the long run [26]. Such practices have been shown to spuriously enhance the likelihood of proving a wrong hypothesis, can provide unfair advantage to few researchers over others, can distort scientific evidence and serve a poor example for young researchers [24]. 

Unfortunately, research culture in low income countries like Pakistan is still in its infancy, focussed mainly to complete mandatory publication quota for promotion and improve resume [27]. The number of publications by medical and dental faculty in Pakistan has increased 5–7 times in the last 20 years due to the obligatory number of publications per faculty member implemented by Pakistan Medical and Dental Council and higher number of approved medical journals by Higher Education Commission [28]. The increase in number, however, does not certify the responsible conduct of researchers per se. This study aims to investigate the questionable research practices amongst faculty members of medical and dental colleges in Pakistan, which in turn may also help raise awareness of QRPs in the research community.

## Methods

A descriptive online survey was conducted from June-August 2022 to determine the QRPs amongst faculty members of medical and dental colleges of Pakistan. Ethical approval was obtained from Ethical Review Committee of Azra Naheed Dental College, Lahore (ANDC/RAC/210/12/01).

Questionnaire: A 43-item pre-validated anonymous questionnaire, based on previous studies assessing research misconduct, was utilized [23]. The questionnaire was also validated by national experts (n = 5) to ensure the items were realistic and relevant to Pakistani health professionals. Experts were provided with an ‘Expert Validation Form’ and were asked about any faults in language, confusing or misleading statements, ease of item understanding, contextual representativeness of each item and any evidence of bias against gender/race/ethnicity/different groups of respondents. Responses with > 80% consensus were accepted. All items were accepted by local experts, and the final questionnaire was piloted on faculty members (*n* = 10) and checked for internal reliability through Cronbach’s alpha. The questionnaire demonstrated good reliability (α = 0.758).

The online questionnaire provided a brief introduction to the research topic and sought informed consent. Upon the participant stating ‘Yes’ to the consent form, the next part inquired about participant demographics like age, gender, discipline of study (Basic sciences/Clinical sciences), profession (Medical / Dental), academic rank (Lecturer, Assistant Professor, Associate Professor, Professor) and the number of publications. The final part investigated QRPs in four domains: Data collection and storage, Data analysis, Study reporting, Collaboration and authorship.

The respondents were asked to choose from “Never”, “Once”, “Occasionally”, “Sometimes” “Frequently” and “Almost always”. Only fulltime faculty members with postgraduate qualifications and working in various disciplines in private/public institutes were invited. Faculty members were initially selected as a ‘curated sample’ by identifying them from official medical and dental institute website. In the next round, ‘virtual snowball sampling’ was done by requesting few faculty members to post questionnaire links on Facebook and WhatsApp faculty groups. The data collection stopped when we did not get further responses after three reminders. To ensure anonymity and encourage honest responses, the names of participants and their institutional names were not recorded. Mandatory item scoring was implemented to avoid missed data.

The data were analysed using IBM SPSS statistical software, version 24 and Microsoft Excel 2013. Descriptive statistics were calculated (Frequencies, Percentages, Mean, SD). To improve readability, the response options of “occasionally,” “sometimes,” “frequently,” and “almost always” were collapsed into a single frequency option labelled “more than once”. Subsequently, each category was given a score as ‘0’ for ‘Never’, ‘1’ for ‘Once and ‘3’ for ‘More than once’. Mean was calculated for each domain. The frequency of respondents above and below the mean value was also calculated. The maximum and minimum possible score for each domain was as follows:

- Data collection and storage: 9 items (min: 0, max: 27).
- Data analysis: 13 items (min: 0, max: 39).
- Study reporting: 14 items (min: 0, max: 42).
- Collaboration and authorship: 7 items (min: 0, max: 21).

Chi Square test was used to analyse association of participants characteristics with each Questionable Research Practices domain. *P*-value < 0.05 was considered statistically significant.

## Results

A total of 654 faculty members responded. The respondents included faculty members from diverse age, gender, disciplines and academic ranks. 95% of the respondents had published manuscripts, while others had completed their research dissertations. Majority of our participants had 10 or less publications (Table 1).

**Table 1:** Participant characteristics

Characteristics		No. (%)
Gender (*n* = 654)	Male	324(49.5)
	Female	330(50.5)
Age (*n* = 654)	26–35 Yrs.	213(32.6)
	36–45 Yrs.	291(44.5)
	46–55 Yrs.	129(19.7)
	56–65 Yrs.	21(3.2)
Discipline of study (*n* = 654)	Basic Sciences	378(57.8)
	Clinical Sciences	276(42.2)
Academic Rank (*n* = 654)	Lecturer	63(9.6)
	Assistant Professor	313(47.9)
	Associate Professor	151(23.1)
	Professor	127(19.4)
Publications (*n* = 654)	None	33(5)
	1–5	216(33)
	6–10	240(36.7)
	11–15	102(15.6)
	16–20	30(4.6)
	Above 20	33(5)

Every respondent reported committing at least one QRP in their career. The most common QRPs included deliberately failing to mention funding, publishing program evaluation data which was not collected for research purposes or approved by an ethical body, inappropriately storing identifiable information, non-disclosure of any conflicts. (Table 2)

**Table 2:** Frequency of questionable research practices

Questionable (Research Practices)		No. (%)					
		More than once		Once		Never	
Data collection and storage	1. Conducted a human-subjects research study without ethics approval (i.e., without Institutional Review Board [IRB] approval)	396	61%	198	30%	60	9%
	2. Circumvented one or more aspects of human-subjects ethics rules (i.e., IRB rules)	507	78%	66	10%	81	12%
	3. Collected course or curriculum data under the guise of “program evaluation” without human subjects’ ethics (IRB) approval with the ultimate intent of using the data for research purposes	582	89%	33	5%	39	6%
	4. Inappropriately stored sensitive research data (e.g., data that contains personally identifiable information)	576	88%	63	10%	15	2%
	5. Inappropriately emailed sensitive research data (e.g., data that contain personally identifiable information)	456	70%	123	19%	75	11%
	6. Stopped collecting data earlier than planned because the results already reached statistical significance, without formal stopping rules	306	47%	240	37%	108	17%
	7. Fabricated data	447	68%	156	24%	51	8%
	8. Pressured a student or other subordinate to be a study participant in your research	507	78%	66	10%	81	12%
	9. Used students or residents as research subjects without informing the overseeing dean, program director, or other pertinent official	467	71.4	125	19.1	62	9.4
Data analysis	10. Deleted data before performing data analysis without disclosure	24	4%	111	17%	519	79%
	11. Ignored a colleague’s use of flawed data	105	16%	114	17%	435	67%
	12. Ignored a colleague’s questionable interpretation of data	108	17%	105	16%	441	67%
	13. Reported a downwardly rounded *p*-value (e.g., reporting that a *p*-value of 0.054 is less than 0.05)	162	25%	141	22%	351	54%
	14. Misrepresented a participant’s words or writings	51	8%	84	13%	519	79%
	15. Decided whether to exclude non-outlier data after looking at the impact of doing so on the results	63	10%	69	11%	522	80%
	16. In a qualitative study, failed to report disconfirming examples or cases that weaken your conclusions	550	84%	91	14%	13	1.9%
	17. Collected more data after seeing that the results were almost statistically significant	459	70%	105	16%	87	13%
	18. To confirm a hypothesis, selectively deleted or changed data after performing data analysis	48	7%	63	10%	543	83%
	19. Reported an unexpected finding as having been hypothesized from the start	81	12%	102	16%	471	72%
	20. Concealed results that contradicted your previous findings or convictions	36	6%	78	12%	540	83%
	21. Claimed you used a particular qualitative research approach appropriately (e.g., grounded theory) when you knowingly did not	549	84%	66	10%	39	6%
	22. Claimed you used a particular qualitative research technique appropriately (e.g., saturation, triangulation) when you knowingly did not	536	82%	104	16%	14	2%
Study reporting	23. Spread study results over more papers than is appropriate (“salami slicing”)	555	85%	60	9%	39	6%
	24. Deliberately failed to mention important limitations of a study in the published paper	531	81%	69	11%	54	8%
	25. Deliberately failed to mention an organization that funded your research in the published paper	594	91%	39	6%	21	3%
	26. Inappropriately modified the results of a study due to pressure from a research adviser or other collaborator	18	3%	51	8%	585	89%
	27. Inappropriately modified the results of a study due to pressure from a funding agency	9	1%	24	4%	621	95%
	28. Failed to disclose relevant financial or intellectual conflicts of interest	573	88%	48	7%	33	5%
	29. Used someone else’s ideas without their permission or proper citation	534	82%	84	13%	36	6%
	30. Used sections of text from another author’s copyrighted material without permission or proper citation	528	81%	84	13%	42	6%
	31. Used sections of text from your own publications without proper citation (“self-plagiarism”)	477	73%	126	19%	51	8%
	32. Selectively cited certain papers just to please editors or reviewers	468	72%	117	18%	69	11%
	33. Cited articles and or materials that you have not read	312	48%	114	17%	228	35%
	34. Selectively cited your own work just to improve your citation metrics	420	64%	132	20%	102	16%
	35. Reused previously published data without disclosure (“duplicate publication”)	27	4.1%	228	34.8%	399	61%
	36. Used confidential information obtained as a reviewer or editor for your own research or publications	22	3.3%	248	38%	384	5.8%
Collaboration and authorship	37. Refused to share data with legitimate colleagues	385	59%	45	7%	224	34%
	38. Added one or more authors to a paper who did not qualify for authorship (“honorary authorship”)	523	80%	106	16%	25	3.8%
	39. Accepted authorship for which you did not qualify (“honorary authorship”)	550	84%	98	15%	6	0.9%
	40. Demanded authorship for which you did not qualify (“honorary authorship”)	324	50%	210	32%	120	18%
	41. Omitted a contributor who deserved authorship	561	86%	75	11%	18	3%
	42. Submitted (or re-submitted) a manuscript or grant application without consent from one or more of the authors	570	87%	60	9%	24	4%
	43. Submitted the same manuscript to multiple journals at once (“duplicate” or “double submission”)	546	83%	102	16%	6	1%

Age, gender and academic rank showed significant association with ‘data collection and storage’ domain score as well as for ‘data analysis’ domain. Higher frequency of above average scores were seen in participants in age group 26–35 and 36–45 years, among females and participants with assistant professor rank. For ‘study reporting’ domain age, gender, academic rank and primary areas of study showed significant association for below and above average scoring categories. However, the ‘collaboration and authorship’ domain showed significant association with age only (Table 3).

**Table 3:** Association of QRPs with demographics

	Data collection and Storage				Data Analysis				Study Reporting				Collaboration and authorship			
	14.61 ± 2.54				29.88 ± 5.65				25.42 ± 5.95				16.93 ± 3.51			
	Below Average		Above Average		Below Average		Above Average		Below Average		Above Average		Below Average		Above Average	
	n	%	n	%	n	%	n	%	n	%	n	%	n	%	n	%
**Age**																
26–35	51	20.7%	162	39.7%	70	29.0%	143	34.6%	73	29.0%	140	34.8%	49	25.4%	164	35.6%
36–45	143	58.1%	148	36.3%	125	51.9%	166	40.2%	134	53.2%	157	39.1%	107	55.4%	184	39.9%
46–55	49	19.9%	80	19.6%	40	16.6%	89	21.5%	42	16.7%	87	21.6%	37	19.2%	92	20.0%
56–65	3	1.2%	18	4.4%	6	2.5%	15	3.6%	3	1.2%	18	4.5%	0	0.0%	21	4.6%
***p*** **-value**	0.000*				0.035*				0.000*				0.000*			
**Gender**																
Male	144	58.5%	180	44.1%	138	57.3%	186	45.0%	144	57.1%	180	44.8%	103	53.4%	221	47.9%
Female	102	41.5%	228	55.9%	103	42.7%	227	55.0%	108	42.9%	222	55.2%	90	46.6%	240	52.1%
***p*** **-value**	0.000*				0.003*				0.002*				0.205			
**Current Academic Rank / Title**																
Lecturer	20	8.1%	43	10.5%	32	13.3%	31	7.5%	22	8.7%	41	10.2%	19	9.8%	44	9.5%
Assistant Professor	106	43.1%	207	50.7%	98	40.7%	215	52.1%	112	44.4%	201	50.0%	87	45.1%	226	49.0%
Associate Professor	73	29.7%	78	19.1%	60	24.9%	91	22.0%	81	32.1%	70	17.4%	56	29.0%	95	20.6%
Professor	47	19.1%	80	19.6%	51	21.2%	76	18.4%	37	14.7%	90	22.4%	31	16.1%	96	20.8%
***p*** **-value**	0.017*				0.015*				0.000*				0.102			
**Discipline of study**																
Basic Sciences	149	60.6%	229	56.1%	143	59.3%	235	56.9%	161	63.9%	217	54.0%	122	63.2%	256	55.5%
Clinical Sciences	97	39.4%	179	43.9%	98	40.7%	178	43.1%	91	36.1%	185	46.0%	71	36.8%	205	44.5%
***p*** **-value**	0.265				0.543				0.013*				0.070			

## Discussion

In this study, we observed the frequency of questionable research practices among medical and dental faculty members of Pakistan. We aim to raise awareness about the practices that could have detrimental effects on the scientific knowledge. To the best of our knowledge, this is the first study conducted to explore the research practices of medical and dental faculty members in South-East Asian region.

Majority of our participants had less than or equal to 10 publications in their lifetime. The reason for this could be attributed to the large number of lecturers and assistant professors in the group who are considered early level researchers. Moreover, our sample included faculty of medical and dental colleges that follow Pakistan Medical & Dental Council (PMDC) regulations. Until recently, these regulations only required 5–8 publications for eligibility as a professor. The recent regulations of PMDC and Higher Education Commission (HEC) Pakistan requires 15 publications for eligibility as a professor. However, these regulations do not prescribe the Journal Impact Factor or Quartile. A study conducted on communications and sociology and other social sciences department revealed that their assistant professors published more manuscripts than their professors [29–32]. In Health Professionals, the number of publications increase with the increase in their academic rank [33–35]. Junior faculty members are hired based on their postgraduate qualifications in Pakistan. The current expectations of ‘Publish or Perish’ from health professionals along with teaching, supervision and provision of clinical services is challenging. Such policies may have forced health professionals toward questionable research practices. Therefore, we recommend multiple streams in faculty promotion regulations with varying criteria for those involved in teaching and scholarship, teaching and research, clinical services and teaching, clinical services and research.

Our results reveal that 57.8% of research publications are from Basic Sciences department. In South-East Asian countries, the clinical faculty is involved in teaching as well as the provision of clinical services to the patient in busy tertiary care teaching hospitals. As the same clinical faculty is expected to teach undergraduate students and treat patients, this may have impacted their ability to conduct research. A survey in India reported that clinical faculty does not have enough time to conduct research while managing their patients at the same time [36]. Practicing doctors in Brazil reported that they do not have sufficient knowledge about research methodology due to lack of formal training, which is why they are less productive in research [37, 38]. In Nepal, only 48% of HPEs had ever published a research paper because they were not aware of the research databases [39]. Research is not taught in undergraduate or postgraduate curriculum, thus health professionals crumble under pressure of publication once they are hired as faculty members.

It has been noted that 100% of our participants have engaged in QRP at least once in their career. The study conducted by Artino disclosed 90.3% HPEs self-reported at least one QRP [23]. A systematic review revealed that 91% researchers in business and psychology were involved in QRPs [17, 40]. A study on sociology researchers in Italy reported 88% participants committing QRP [41]. It is important to highlight that unethical research practices are different from irresponsible research practices, although, their impact on the integrity of scientific knowledge may be severe. It was only in 2012, that researchers published a list of QRPs to highlight the factors violating good scientific practice [42]. Although gifting honorary authorships, salami slicing data or excluding study limitations seem less criminal in nature, may have more ambiguous and grave consequences because of the high frequency of such actions. The problem of QRP is a global issue and not just limited to health professionals. However, in countries like Pakistan which lack monitored ethical bodies, these results are not a surprise.

There was significant association of age, gender and academic rank with ‘Data collection and storage’ and ‘Data Analysis’ domains. Young researchers, female faculty members and early level faculty members have higher QRPs than others. Researched published without proper Ethical Review Board approval is not a new case. In USA, studies have been published unethically about minority groups in the past [43], which is why they underwent several reforms to promote IRB approval system [44–46]. Sadly, we do not find such reforms in our contextual literature. There is a stigma attached to this sensitive topic which pushes researchers to brush such issues under the carpet instead of publishing them and urge for reforms. A letter to editor was published in Nature Medicine explaining that in Pakistan, like other African and Asian countries, does not possess a regulatory body to monitor ethics in clinical research. Each institution sets up its own IRB team without formally trained professionals. ‘The Centre of Biomedical Ethics and Culture’ in Karachi and ‘Pakistan Medical Research Council’ initiated training programs for faculty members but their approach is limited and have not been able to contribute substantially in the field of research [47]. 

Unlike other countries, there is no trend of depositing research data in online certified repositories which renders the data collection method questionable. Although Pakistan does not contain a data repository of its own, the Global Health Observatory Data Repository of WHO is a good platform to submit legitimate data. Similarly, many journals encourage authors to submit raw data to secured repositories like the Harvard Dataverse, Open Science Framework, NHS digital etc. which help transparent data collection and data storage [48, 49]. A study conducted on researchers of Saudi Arabia, Jordan and Egypt reported that 60.1% of the researchers never used metadata standards because they were unaware of such facilities (20%), lack of institutional support and time (33.4%) and not knowing the importance of data sharing (35%) [50]. 

QRP in ‘Collaboration and authorship’ domain was also observed in our study. In Pakistan, for the last two years, the number of publications required for the promotion have been tripled [51]. Some of the institutions have made their own policies regarding articles acceptance for promotions. There is no “W” category (high impact) medical journal in Pakistan so far, and research facilities are also not well developed for high quality research, yet the institutions are demanding high quality impact factor publications. To achieve this, young researchers indulge in misconduct. A survey was conducted in Pakistan in which 67% of the researchers considered poor training and lack of awareness as barriers to conduct research [52]. Another publication highlighted multiple questionable practices of authors in a Pakistani journal namely plagiarism, duplicate publication, writing supervisor’s name as the first author and gift authorships to non-contributors [53]. 

A complete discussion of all factors may not be possible within the scope of this paper. However, we aim to emphasize on the helm of affairs which are not less than ‘alarming’ in nature. The results of our study may be transferable to other South-East Asian and African countries which lack effective regulatory bodies to ensure ethical and responsible research. Our study has several limitations, firstly, it is a self-reported questionnaire, and our findings may not be generalisable. Given the sensitive nature of the topic, some may have chosen not to provide answers or given responses that were socially acceptable. Similarly, we did not collect data from other health professionals like pharmacists, nurses etc. Nonetheless, we were able to exhibit the existence of QRPs in our medical and dental faculty members. This paper may help raise awareness of the situation and motivate researchers to avoid QRPs in future.

## Conclusion

The medical and dental faculty members participating in this study reported involvement in a range of questionable research practices (QRPs). Their confession might have contributed to the faculty developing self-awareness and reinforcing academic integrity. It is imperative to introduce reforms in research practices in order to uplift scientific integrity and literary culture in low-income countries. Future research should explore the factors resulting in such practices and improve research culture.

## Data Availability

The datasets generated and/or analysed during the current study are not publicly available due to ethical guidelines and sensitive nature of the data.
